# Investigation of the Genes Involved in Antigenic Switching at the *vlsE* Locus in *Borrelia burgdorferi*: An Essential Role for the RuvAB Branch Migrase

**DOI:** 10.1371/journal.ppat.1000680

**Published:** 2009-12-04

**Authors:** Ashley R. Dresser, Pierre-Olivier Hardy, George Chaconas

**Affiliations:** 1 Department of Biochemistry & Molecular Biology, The University of Calgary, Calgary, Alberta, Canada; 2 Department of Microbiology & Infectious Diseases, The University of Calgary, Calgary, Alberta, Canada; Medical College of Wisconsin, United States of America

## Abstract

Persistent infection by pathogenic organisms requires effective strategies for the defense of these organisms against the host immune response. A common strategy employed by many pathogens to escape immune recognition and clearance is to continually vary surface epitopes through recombinational shuffling of genetic information. *Borrelia burgdorferi*, a causative agent of Lyme borreliosis, encodes a surface-bound lipoprotein, VlsE. This protein is encoded by the *vlsE* locus carried at the right end of the linear plasmid lp28-1. Adjacent to the expression locus are 15 silent cassettes carrying information that is moved into the *vlsE* locus through segmental gene conversion events. The protein players and molecular mechanism of recombinational switching at *vlsE* have not been characterized. In this study, we analyzed the effect of the independent disruption of 17 genes that encode factors involved in DNA recombination, repair or replication on recombinational switching at the *vlsE* locus during murine infection. In *Neisseria gonorrhoeae*, 10 such genes have been implicated in recombinational switching at the *pilE* locus. Eight of these genes, including *recA*, are either absent from *B. burgdorferi*, or do not show an obvious requirement for switching at *vlsE*. The only genes that are required in both organisms are *ruvA* and *ruvB*, which encode subunits of a Holliday junction branch migrase. Disruption of these genes results in a dramatic decrease in *vlsE* recombination with a phenotype similar to that observed for lp28-1 or *vls-*minus spirochetes: productive infection at week 1 with clearance by day 21. In SCID mice, the persistence defect observed with *ruvA* and *ruvB* mutants was fully rescued as previously observed for *vlsE*-deficient *B. burgdorferi*. We report the requirement of the RuvAB branch migrase in recombinational switching at *vlsE*, the first essential factor to be identified in this process. These findings are supported by the independent work of Lin et al. in the accompanying article, who also found a requirement for the RuvAB branch migrase. Our results also indicate that the mechanism of switching at *vlsE* in *B. burgdorferi* is distinct from switching at *pilE* in *N. gonorrhoeae*, which is the only other organism analyzed genetically in detail. Finally, our findings suggest a unique mechanism for switching at *vlsE* and a role for currently unidentified *B. burgdorferi* proteins in this process.

## Author Summary

A common strategy for evasion of the host immune system is the continuous variation of a major surface protein that elicits a dominant immune response (antigenic variation). Many pathogens accomplish this goal by unidirectional movement of DNA sequence information from silent or archival gene copies into an expression site. The molecular details of how this gene shuffling is accomplished are not understood for any organism. In the flat-wave shaped bacterium causing Lyme disease, information is moved from 15 silent cassettes into the *vlsE* gene to promote antigenic variation. In this work we have investigated the effect of independent mutation of 17 DNA replication, recombination and repair genes on the movement of genetic information into *vlsE*. We found that mutation of either of the genes encoding the two subunits of the RuvAB branch migrase blocked transfer of genetic information into *vlsE* during mouse infections, identifying the first required function for antigenic variation in the Lyme disease spirochete.

## Introduction

Antigenic variation through targeted genome rearrangements is a common strategy for immune evasion and has been identified in many important pathogens including protozoa [1],[2],[3],[4], bacteria [5],[6],[7],[8],[9],[10],[11] and fungi [12]. In spite of the common occurrence of this strategy for immune evasion amongst pathogens, few molecular details of the recombinational switching processes that generate diversity in antigen-expressing genes have been reported for any organism.

Lyme borreliosis is a world wide health problem. It is a multisystemic illness caused by the spirochete *Borrelia burgdorferi*, and related species. Disease progression occurs through three stages: early, disseminated and persistent and can result in various arthritic, cardiac and neurological concerns if left untreated [13],[14],[15]. Persistent infection by *B. burgdorferi* requires continual segmental gene conversion at the *vlsE* locus, which encodes a 35 kDa membrane lipoprotein [9],[16],[17],[18],[19]. The *vlsE* gene, or expression locus is carried at the right end of the linear plasmid lp28-1. In the absence of lp28-1 or when the *vls* locus is deleted a productive murine infection ensues, but the spirochetes are cleared between days 8 and 21 post-infection [16],[17],[20],[21]. Adjacent to *vlsE* (also referred to as *vls1*), is a contiguous upstream array of 15 silent cassettes separated from each other by 17 bp direct repeats, which also flank the *vlsE* variable region (see Fig. 1C in [18]). During murine infection (and probably in other mammals) information is transferred unidirectionally from the silent cassettes into the expression site to generate diversity at six regions (VR1–VR6) within the central region of the *vlsE* gene [17],[18],[19]. These regions correspond to highly exposed regions of the VlsE protein and are believed to be prominently displayed antigenic areas [22]. Generation of antigen diversity occurs through segmental gene conversion such that information from several silent cassettes can be transferred into the single *vlsE* locus to generate a mosaic gene with possibilities for the production of myriad unique VlsE proteins. All silent cassettes are utilized as sequence donors in the gene conversion events at *vlsE* and the majority of recombination events are short, ranging from 1–22 codon changes [17]. Similarly, the requirement for flanking sequence homology is also short, in the neighborhood of approximately 10 nucleotides.

**Figure 1:**
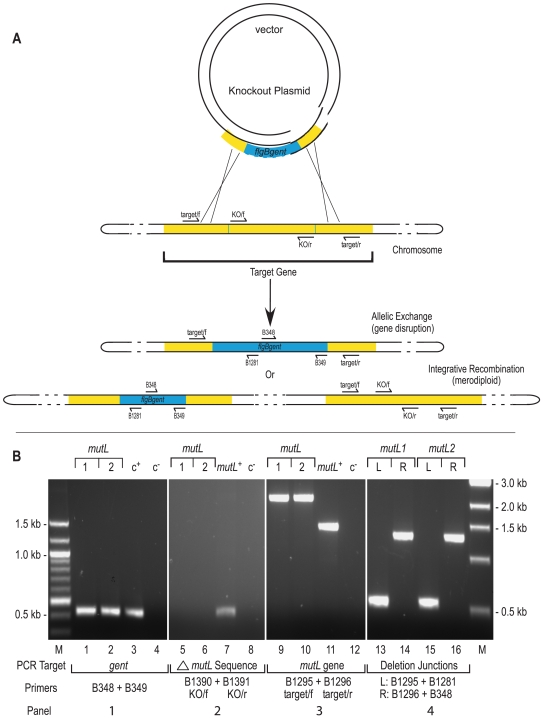
Gene disruption and confirmation. **A)** Gene disruption strategy. The infectious *B. burgdorferi* strain B31, clone 5A4 (B31-5A4) was transformed with a knockout plasmid carrying a one kb gentamicin cassette (blue) that replaced the central portion of the target gene (yellow) as described in Materials and Methods. The two possible outcomes of recombination events with the target gene are shown: allelic exchange would result in gene disruption while integrative recombination of the knockout plasmid would result in merodiploid formation. The position of PCR primers used for construct verification are shown by arrows on the schematic. **B)** Construct verification of the *mutL* disruption by PCR. Each gene disruption was subjected to four PCR analyses. **1)** The presence of the gentamicin resistance cassette was confirmed as shown in lanes 1 and 2. The shuttle vector pBSV2G [71] served as the positive control c^+^ for amplification of the *gent* cassette (lane 3.) **2)** The portion of *mutL* expected to be deleted in a gene disruption was not detected in either *mutL*1 or 2 (lanes 5 and 6); however, it was detected in the positive control (c+), which contained wild-type B31-5A4 DNA as a template in lane 7. **3)** The size of the target gene was compared in *mutL*1 and 2 genotypes. The expected 2.1 kb products for a gene disruption were observed (lanes 9 and 10) in comparison to the 1.5 kb product from the *mutL^+^* genotype (lane 11). Lanes 4, 8 and 12 are negative controls (c^−^) that lacked DNA template. **4)** Confirmation of the correct insertion site was performed using combinations of the target gene primers and primers internal to the gentamicin cassette to amplify the boundaries. The left boundary in both *mutL* clones gave the expected 0.55 kb product (lanes 13 and 15). The right boundary in both clones gave the expected product of approximately 1.3 kb (lanes 14 and 16). A 100bp ladder on the left side, relevant to the two left panels, and a 1kb ladder on the right side, which applies to the two right panels, were the molecular weight markers (M) used.

An interesting feature of switching at *vlsE* is that it does not occur when spirochetes are grown in culture or when they reside in the tick midgut. [18],[23]. Moreover, the acquired immune response is not required, as switching occurs in SCID mice, which lack the ability to mount an acquired immune response to antigenic challenge [9],[16],[17],[20]. The mammalian signal that triggers recombinational switching remains unknown at this time. These features make the study of antigenic variation in *B. burgdorferi* difficult and limit these studies to animal infection models. In the mouse, antigenic switching can be detected four days after infection and by 28 days no parental *vlsE* sequences remain in the population of spirochetes recovered from some tissues in infected animals [9],[17],[20].

Even though *B. burgdorferi* has a small genome [24],[25], genetic manipulation is time consuming, inefficient and sometimes difficult [26]. The protein machinery that promotes recombinational switching at *vlsE* is, therefore, unknown at this time. A single study towards this end has reported that the *B. burgdorferi recA* gene is not required for antigenic switching [27]. In this study we generated 17 mutants carrying disruptions in known DNA recombination, repair and replication genes in the hopes of identifying proteins involved in recombinational switching at *vlsE*. A single recombination function, the RuvAB Holliday junction branch migrase encoded by the *ruvA* and *ruvB* genes, was unambiguously identified as a requirement for switching at *vlsE*, a result also reported in the accompanying paper by Lin et al [28]. In contrast, 10 known recombination, repair, or replication genes are required in recombinational events underlying antigenic switching at *pilE* in *N. gonorrhoeae* [29],[30],[31],[32],[33],[34],[35],[36],[37],[38],[39]. Eight of those genes are either missing or not required for switching at *vlsE* in *B. burgdorferi*. Our results point towards a unique mechanism for switching at *vlsE* in and suggest that it may involve specialized proteins that help to mediate the process.

## Results

### Construction of DNA repair and replication gene disruptions in *B. burgdorferi*

A systematic approach was undertaken to disrupt 21 different genes in order to investigate their role in *vlsE* recombination in *B. burgdorferi*. Knockout plasmids were constructed (**Figure S1**) and used to transform the infectious *B. burgdorferi* B31 clone 5A4 [40]. Following transformation, allelic exchange results in successful gene disruption (**Fig. 1A**). However two other transformation outcomes can arise: integretative recombination, which results in merodiploid formation, and cases where no recombinants are recovered [26]. To investigate the structure of the *B. burgdorferi* transformants, they were screened using PCR with various primer combinations (**Fig. 1B**). The presence of the gentamicin resistance cassette (**Panel 1**) and the absence of the expected deleted sequences (∼500 bp) from the disrupted target gene (**Panel 2**) were first confirmed using the indicated primer sets. The target gene was also amplified (**Panel 3**) to confirm the approximate 0.7kb size increase relative to wild-type DNA due to the insertion of the gentamicin resistance cassette. Finally, the correct insertion site was verified using combinations of the target and knockout primers to amplify the insertion boundaries (**Panel 4**). In addition to the PCR analyses, gene disruptions were independently confirmed by Southern hybridizations using probes specific to the gentamicin resistance cassette and the deleted portion of the target gene (see **Fig. S2** and **Table S2**).

Of the 21 DNA replication, repair and recombination gene knockouts attempted, 17 were successful (**Table 1**). When a disruption attempt was unsuccessful, the knockout plasmid was re-constructed in an effort to minimize possible effects on adjacent gene expression from read-through of transcription from the *gent* cassette. This was accomplished by either changing the polarity of the gentamicin resistance cassette relative to the gene target, or by adding (or removing) a T7 transcriptional terminator. Three gene targets required reconstruction of the knockout plasmid in order to successfully obtain *B. burgdorferi* gene disruptions. *recJ* was first attempted without the T7 terminator in the reverse orientation and resulted only in merodiploids. The gentamicin resistance cassette in the forward orientation with the T7 terminator did result in knockouts and further attempts were halted. The *sbcD* knockout was first attempted with a construct containing the T7 terminator and the gentamicin resistance cassette in the reverse orientation. This attempt resulted only in merodiploids; however, when the polarity was changed to the forward orientation, allelic exchange was successful. The *recA* disruption was also difficult to obtain. Unsuccessful attempts were first made with the gentamicin resistance cassette in the forward orientation with and without the T7 terminator. When the T7 terminator was removed and the *gent* gene was in the reverse polarity, true knockouts of the *recA* gene were obtained. Difficulty in obtaining a *recA* gene disruption has also been previously reported [41], however, a single *recA* null mutant has been previously constructed with the insertion of a kanamycin resistance cassette in the forward orientation [27]. Finally, *dnaB*, *hbb*, *recB* and *recC* knockouts were not obtained despite changing the polarity of the gentamicin resistance cassette and adding or removing a T7 transcriptional terminator.

**Table 1:** Gene disruption targets and knockout plasmid attributes.

Gene target	Locus	Gene description	Plasmid	*E. coli* strain number (GCE)	Polarity of gent relative to target	T7 terminator	Gene disruption	Merodiploid
*recJ*	BB0254	ssDNA-specific exonuclease	pAD51pAD26	15631538	forwardreverse	+−	+−	−+
*priA*	BB0014	helicase	pAD94	1908	forward	+	+	−
*sbcD*	BB0829	exonuclease	pAD87pAD86	15991598	forwardreverse	++	+−	−+
*ruvA*	BB0023	Holliday junction helicase	pAD78	1590	forward	+	+	−
*mutL*	BB0211	mismatch repair protein	pAD61	1573	forward	+	+	−
*ruvB*	BB0022	Holliday junction helicase	pPOH6	1618	forward	−	+	−
*sbcC*	BB0830	exonuclease	pAD65	1577	forward	+	+	−
*BBG32*	BBG32	putative helicase	pAD88	1900	forward	+	+	−
*mutS1*	BB0797	mismatch repair protein	pPOH2	1604	forward	−	+	−
*mutS2*	BB0098	mismatch repair protein	pAD24	1536	reverse	−	+	−
*recA*	BB0131	DNA-dependent ATPase	pAD101pAD92pAD102	191319041914	reverseforwardforward	−+−	+−−	−−−
*recG*	BB0581	ATP-dependent helicase	pAD49	1561	reverse	+	+	−
*rep*	BB0607	ssDNA-dependent ATPase helicase	pAD53	1565	reverse	+	+	−
*nucA*	BB0411	exonuclease involved in competency	pAD63	1575	forward	+	+	−
*mag*	BB0422	3′-methyladenine DNA glycosylase	pAD57	1569	forward	+	+	−
*mfd*	BB0623	transcription-repair coupling factor	pAD59	1571	reverse	+	+	−
*nth*	BB0745	endonuclease III	pPOH28-5	1684	reverse	−	+	−
*dnaB*	BB0111	replicative helicase	pAD80pAD81	15921593	forwardreverse	++	−−	−−
*hbb*	BB0232	DNA-binding protein	pAD100pAD99pAD106	191219111918	reverseforwardreverse	++−	−−−	−−−
*recB*	BB0633	exonuclease	pAD22pAD48	15341560	reversereverse	−+	−−	−−
*recC*	BB0634	exonuclease	pAD84pAD104pAD103	159619161915	reverseforwardreverse	+−−	−−−	−−−

### Effect of *B. burgdorferi gene* disruptions on C3H/HeN mouse infections: an essential role for *ruvAB* in recombinational switching

For each gene disruption two clones were chosen which contained the full plasmid complement required for infectivity, as determined by PCR screening with primers specific to the plasmids. Most mutant constructs contained the full complement of plasmids found in the parental clone B31 5A4 [40]. Some of the mutant *B. burgdorferi* clones lacked plasmids that are not required for infection or persistence as follows: *recJ*1 lacks lp28-2, *mag2* lacks lp28-4, *ruvA*1 is missing cp9, *mutL*2 lacks cp9 and cp32-3, *nucA*1 lacks lp21 and cp32-3, *ruvB5* lacks lp28-4 and cp9 and *priA*3, *recA*2 and *recA*3 are missing cp9. The possible effect of the mutations on growth of *B. burgdorferi* in culture was assessed by performing growth comparisons of each of the mutants with the wild-type clone 5A4. All mutants displayed growth curves that were indistinguishable from the parent strain (data not shown).

**Figure 2:**
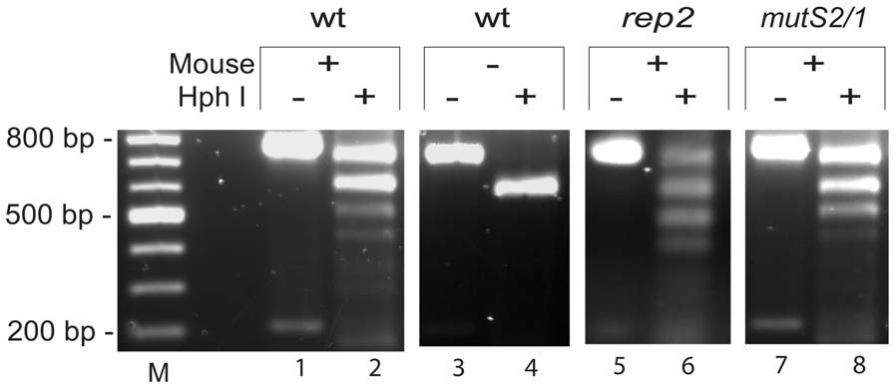
Restriction fragment length polymorphism assay for switching at *vlsE*. A portion of the *vlsE* expression site containing the variable regions was amplified using primers B248 and B249 to give a product of 776 bp. PCR reactions were performed on *B. burgdorferi* grown from ear biopsies taken at day 21 and the products were digested with HphI and run on a 1.2% agarose gel in TAE buffer at 75V for 1.5 hours and stained with ethidium bromide (see Materials and Methods). Wild-type *B. burgdorferi* B31-5A4 recovered following infection of a C3H/HeN mouse was used as a template in lanes 1 and 2. An unswitched template (not exposed to mouse infection) is shown in lanes 3 and 4. PCR products from *rep2* and *mutS2*/1 DNA templates are found in lanes 5 & 6, and 7 & 8 respectively. M denotes a 100bp molecular weight marker.

Two independent knockout clones for each mutation were used to infect C3H/HeN immunocompetent mice (at least two mice for each clone, four mice for each mutated gene) as described in Materials and Methods. Cultures were grown from blood samples at day 7 to monitor infectivity. At days 14 and 21, by which time spirochetes are largely cleared from the blood, ear biopsies were used to monitor infection and switching at *vlsE*. The upper portion of **Table 2** shows mutant strains that displayed a productive infection (≥75% of mice infected at day 7) that did not decline at day 21, the point post-infection when strains unable to switch at *vlsE* have been cleared [16],[17],[20],[42]. The variable region of *vlsE* was amplified from DNA isolated from day 21 ear cultures and analyzed using RFLP assays (**Fig. 2**), which detect new restriction sites resulting from switching at the *vlsE* locus [16]. As noted in the upper portion of **Table 2**, *mutS2*, *recA*, *recG*, *rep*, *nucA*, *mag*, *mfd* and *nth* mutants all displayed switching at the *vlsE* locus.

**Table 2:** Effect of DNA repair and replication mutants on *B. burgdorferi* inefction and switching at *vlsE* in C3H/HeN mice.

*B. burgdorferi* genotype	Strain (GCB)	Total mice^a^	Day 7 Blood^b^	Day 7 Infection	Day 21 Ear	Day 21 Infection	Switching at *vlsE* day 21^c^							
5A4 wt	933	18	18/18	100.0%	18/18		+							
*mutS2/1 (BB0098) mutS2/2*	1135 1136	4	2/2 2/2	100.0%	2/2 2/2	100.0%	+							
*recA2 (BB0131) recA3*	1284 1285	4	2/2 2/2	100.0%	2/2 2/2	100.0%	+							
*recG1 (BB0581) recG2*	1155 1156	4	2/2 2/2	100.0%	2/2 2/2	100.0%	+							
*rep1 (BB0607) rep2*	1158 1159	4	2/2 2/2	100.0%	2/2 2/2	100.0%	+							
*nucA1 (BB0411) nucA2*	1176 1177	4	2/2 1/2	75.0%	2/2 2/2	100.0%	+							
*mag1 (BB0422) mag2*	1161 1162	4	2/2 1/2	75.0%	2/2 1/2	75.0%	+							
*mfd1 (BB0623) mfd2*	1180 1181	4	2/2 1/2	75.0%	2/2 1/2	75.0%	+							
*nth*1 (BB0745) *nth*2	525 526	4	2/2 2/2	100.0%	2/2 2/2	100.0%	+							
								**Day 35**					**Persistence at day ≥35**	**Switching at ** ***vlsE*** ** day 35** f
								**Heart**	**Bladder**	**Joint**	**Ear**	**Total sites** e		
5A4 wt^d^	933							4/4	4/4	4/4	4/4	16/16	100.0%	+
*recJ1 (BB0254) recJ5*	1153 1154	4	2/2 2/2	100.0%	0/2 0/2	0%	n/a	2/2 1/2	2/2 1/2	0/2 1/2	2/2 2/2	6/8 5/8	68.8%	+
*ruvB4 (BB0022) ruvB5*	513 514	4	2/2 2/2	100.0%	0/2 0/2	0%	n/a	0/2 0/2	0/2 1/2	0/2 1/2	0/2 0/2	0/8 2/8	12.5%	−g
*ruvA1 (BB0023) ruvA2*	1174 1175	4	2/2 2/2	100.0%	0/2 0/2	0%	n/a	0/2 1/2	1/2 0/2	0/2 1/2	1/2 0/2	2/8 2/8	25.0%	−g
*sbcD1 (BB0829) sbcD2*	1251 1252	4	1/2 1/2	50.0%	0/2 1/2	25.0%	+	1/2 1/2	1/2 1/2	1/2 1/2	1/2 1/2	4/8 4/8	50.0%	+
*sbcC2 (BB0830) sbcC3*	1248 1249	4	2/2 0/2	50.0%	2/2 0/2	50.0%	+	2/2 0/2	2/2 0/2	2/2 0/2	2/2 0/2	8/8 0/8	50.0%	+
*BBG32/6 BBG32/7*	1233 1234	4	0/2 2/2	50.0%	0/2 2/2	50.0%	+	0/2 2/2	0/2 2/2	0/2 2/2	0/2 2/2	0/8 8/8	50.0%	+
*priA2 (BB0014) priA3*	1205 1206	4	1/2 0/2	25.0%	0/2 0/2	0%	n/a	1/2 2/2	1/2 2/2	1/2 2/2	1/2 2/2	3/8 8/8	68.8%	+

*B. burgdorferi* genotype	Strain (GCB)	Total mice^a^	Day 7 Blood^b^	Day 7 Infection	Day 21 Ear	Day 21 Infection	Switching at *vlsE* day 21^c^	Day 35					Persistence at day ≥35	Switching at *vlsE* day 35^f^
								Heart	Bladder	Joint	Ear	Total sites^e^		
*mutL1 (BB0211) mutL2*	1178 1179	4	0/2 0/2	0.0%	0/2 0/2	0%	n/a	0/2 1/2	0/2 1/2	0/2 1/2	0/2 1/2	0/8 4/8	25.0%	+
*mutS1/3 (BB0797) mutS1/9*	502 504	6	1/3 3/3	66.7%	1/3 3/3	66.7%	n/a	1/3 2/3	1/3 2/3	1/3 3/3	1/3 3/3	4/12 10/12	58.3%	+

a Total number of mice analyzed.

b Values listed correspond to number of cultures positive/number of sites tested.

c Switching was determined by RFLP assay from available ear cultures at day 21.

d Four mice infected with *B. burgdorferi* 5A4 were chosen as positive controls for organ harvests at day 35.

e Number of positive tissue sites/number of sites tested.

f Switching was determined by RFLP assay from available organ harvest cultures at day 35.

g Switching by RFLP was negative, but DNA sequencing revealed low frequency switching at *vlsE* in all cases.

The lower portion of **Table 2** shows mutant strains that displayed <75% positive cultures from ear biopsies at day 21. When spirochetes could be cultivated at day 21 (*sbcD*, *sbcC* and *BBG32*) switching was monitored and shown to occur using the RFLP assay. For the remainder of the mutant strains, infections were allowed to continue until day 35. At this time the mice were euthanized and spirochetes cultivated from heart, bladder, joint and ear. RFLP switching assays indicated that switching at *vlsE* had occurred in all mutant strains from tissues where spirochetes could be recovered at day 35, with the exception of those carrying the *ruvA* or *ruvB* mutations, whose functional genes encode the two subunits for a Holliday junction branch migrase [43],[44],[45]. Although the *ruvA* and *ruvB* mutant strains recovered from organ harvest at day 35 were negative for switching by RFLP, DNA sequencing analysis revealed that switching had occurred at low efficiency (data not shown).

### Effect of *B. burgdorferi* gene disruptions on SCID C3H/HeN mouse infections

Mutants of *B. burgdorferi* strains that did not show switching in wild-type C3H/HeN mice using the RFLP assay (*ruvA* and *ruvB*) and five other mutants that displayed a decreased persistence at day 21 (*recJ*, *mutL*, *sbcC*, *sbcD* and *BBG32*) were used to infect SCID C3H/HeN mice, which lack an acquired immune response. This effectively removes the selective pressure on antigenic variation and allows *B. burgdorferi* mutants with defective switching at *vlsE* to persist in the host. Direct analysis of *vlsE* switching beyond 21 days post-infection can, therefore, be performed in SCID mice, whereas by this time non-switching spirochetes would be cleared in a wild-type mouse [16],[17],[20],[42]. All the mutant strains tested displayed wild-type levels of infectivity and persistence throughout the 35 day course of infection in SCID mice (**Table 3**). This indicated that the *ruvA* and *ruvB* mutant strains, which did not switch in wild-type mice using the RFLP assay and which showed greatly reduced levels of persistence at day 21 (**Table 2**, bottom), were fully competent for the infection process in mice lacking an acquired immune response. The other mutant *B. burgdorferi* strains that displayed reduced infectivity and persistence were also fully rescued in mice lacking an acquired immune response.

**Table 3:** Effect of DNA repair and replication mutations on *B. burgdorferi* infection in SCID C3H/HeN mice.

*B. burgdorferi* genotype	Strain (GCB)	Total mice^a^	Day 7 Blood^b^	Day 7 Infection	Day 21 Ear	Day 21 Infection	Day 35					Persistence at day 35
							Heart	Bladder	Joint	Ear	Total sites^c^	
5A4 wt	933	6	6/6	100.0%	6/6	100.0%	6/6	6/6	6/6	6/6	24/24	100.0%
*recJ1 (BB0254)recJ5*	11531154	4	2/22/2	100.0%	2/22/2	100.0%	2/22/2	2/22/2	2/22/2	2/22/2	8/88/8	100.0%
*ruvB4 (BB0022)ruvB5*	513514	4	2/22/2	100.0%	2/22/2	100.0%	2/22/2	2/22/2	2/22/2	2/22/2	8/88/8	100.0%
*ruvA1 (BB0023)ruvA2*	11741175	4	2/22/2	100.0%	2/22/2	100.0%	2/22/2	2/22/2	2/22/2	2/22/2	8/88/8	100.0%
*mutL1 (BB0211)mutL2*	11781179	4	1/21/2	50.0%	2/22/2	100.0%	2/22/2	2/22/2	2/22/2	2/22/2	8/88/8	100.0%
*sbcD1 (BB0829)sbcD2*	12511252	4	2/22/2	100.0%	2/22/2	100.0%	2/22/2	2/22/2	2/22/2	2/22/2	8/88/8	100.0%
*sbcC2 (BB0830)sbcC3*	12481249	4	2/22/2	100.0%	2/22/2	100.0%	2/22/2	2/22/2	2/22/2	2/22/2	8/88/8	100.0%
*BBG32/6BBG32/7*	12331234	4	2/22/2	100.0%	2/22/2	100.0%	2/22/2	2/22/2	2/22/2	2/22/2	8/88/8	100.0%

a Total number of mice examined for genotype.

b Values listed correspond to number of cultures positive/number of sites tested.

c Number of positive tissue sites/number of sites tested.

### Analysis of switching at *vlsE* by DNA sequencing of mutant strains recovered from SCID mouse infections

The RFLP assay used here provides a quick and convenient assay method to detect switching at *vlsE* [16]. The incorporation of new restriction endonuclease sites from the silent cassettes into the variable region of *vlsE* is a clear indicator of the switching process. However, the fact that the assay is not quantitative, coupled with the observation that switching is apparently less frequent in SCID mice [17], led us to further analyze switching in SCID mice in a limited set of mutants by DNA sequencing. We chose the two mutants that were negative for switching in wild-type mice by RFLP analysis (*ruvA* and *ruvB*) as well as two mutants that were shown to switch by RFLP at 35 days in wild-type mice, but that displayed no spirochetes in ear cultures at 21 days (*recJ* and *mutL*).

For DNA sequencing studies the same PCR product used for the RFLP assay (a 776 bp fragment containing the *vlsE* variable region) was amplified from the spirochetes recovered from four different tissue types at day 35 for each SCID mouse and gel purified (see Material and Methods). Equimolar amounts of the *vlsE* PCR product from each tissue from the 4 mice used in the infections were combined, providing four pools for each disrupted gene: heart, bladder, joint and ear. The pools were cloned and 10 *E. coli* clones were chosen for each tissue type for a total of 40 *vlsE* sequences examined for each of the *recJ*, *mutL*, *ruvA*, *ruvB* and wild-type genotypes. Using a primer specific to the cloning vector, the plasmid DNA was sequenced and compared to the *B. burgdorferi* 5A4 parental *vlsE* sequence for both templated (present in a silent cassette) and non-templated nucleotide changes [17],[19]. Each sequenced clone where switching at *vlsE* had occurred displayed a unique sequence; hence, all switch variants from each mouse represented independent switching outcomes.

**Figure 3:**
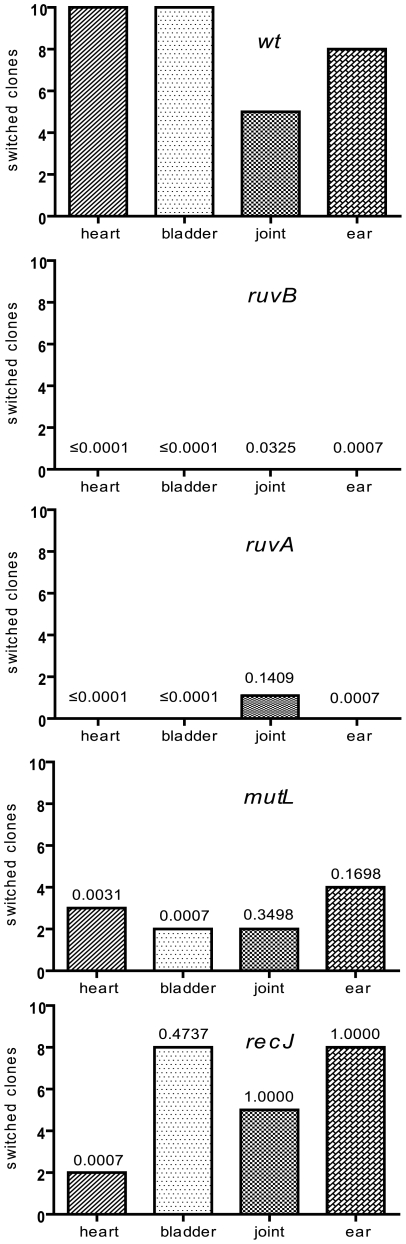
Number of switched *vlsE* clones in SCID C3H/HeN mice. Sequencing of the cloned PCR product of the *vlsE* variable regions using primer pJET1.2/forward was performed on 10 clones from each tissue type culture for each genotype (see Materials and Methods). The y-axis denotes the number of clones out of ten that contained templated nucleotide changes in variable regions 1–6 (switches) and the x-axis denotes the tissue type. The P-values above the bars indicate the level of significance of the difference between the wild-type and mutant samples, calculated using Fisher's Exact test.

Sequencing revealed that 10 out of 10 wild-type clones contained nucleotide changes corresponding to sequences found in the silent cassettes in the heart and bladder tissue cultures while 5/10 and 8/10 clones had switched in the joint and ear tissues, respectively (see **Fig. 3**). These results are similar to previously reported data which indicated a greater proportion of switched clones in heart, bladder and skin tissues than in joint and ear tissues [17]. The overall switching frequency that we observed (82.5% at day 35 post-infection in SCID C3H/HeN mice) also correlates closely with the value of 85% at 28 days post-infection recently observed [17]. For proper analysis, a tissue-specific comparison between mutants and wild-type spirochetes was undertaken as shown in **Fig. 3**.

The most significant reduction in switching occurred with *ruvA* and *ruvB* mutants with only one of 40 clones (2.5%) differing from the wild-type *vlsE* sequence in *ruvA* and no changes observed in any of the 40 *ruvB* clones. The single clone demonstrating switching at *vlsE* in the *ruvA* mutant was from a clone cultivated from joint that displayed at least four exchanges with silent cassettes and did not show any features with obvious differences from switching in wild-type *B. burgdorferi*. The P-values indicated a significant difference (<0.05) in the incidence of switching for all tissues, with the exception of the *ruvA* mutant in joint. These results corroborated the negative switching phenotype of the *ruvA* and *ruvB* mutants observed in the RFLP switching assay after infection of wild-type and SCID mice (**Tables 2** and **3**). The results are further strengthened by the fact that *ruvA* and *ruvB* encode the two subunits of an enzyme known to promote branch migration of Holliday junction recombination intermediates.

Clones carrying a *mutL* mutation displayed an intermediate phenotype with a decrease in switching resulting in a total of only 27.5% of the clones exhibiting nucleotide changes, versus 82.5% for wild-type. Significant tissue-specific differences were observed in the bladder and heart but not the joint and ear (**Fig. 3**). *recJ* showed a slight change in the level of switching with 57.5% of the clones displaying changes in the *vlsE* variable region compared to 85% for wild-type. A significant difference in tissue-specific switching was only observed in the heart. Switching sequencing data were also analyzed by counting the number of nucleotide changes in each clone (**Table S3**) and gave similar results (see also Discussion). DNA sequencing was also performed on the *vlsE* variable region from infections with *sbcC, sbcD* and *BBG32 B. burgdorferi* mutants, which displayed wild-type switching levels in all four (*sbcC*) tissues or in three of the four four (*sbcD* and *BBG32*) tissue types (data not shown).

In addition to the apparent templated nucleotide changes observed in switches at *vlsE*, some non-templated changes (NTCs), where new sequence at *vlsE* did not correspond to the sequence found in any of the silent cassettes, were also observed [17],[46]. There were no NTCs in the total of 80 *vlsE* sequences analyzed for wild-type and *ruvB* clones. In *ruvA*, four NTCs were observed in non-switching clones and three in the single clone that switched. In the *mutL* mutant there were five NTCs and all of these occurred in clones that did not switch. Finally the *recJ* heart sample had six NTCs in three clones, two of which switched. Taken together there were a total of 18 NTCs in the *ruvA*, *mutL and recJ* mutants.

## Discussion

Attempts were made to generate 21 disruptions in *B. burgdorferi* genes believed to be involved in DNA recombination, repair and/or replication to investigate their possible roles in recombinational switching at the *vlsE* locus. Seventeen genes were successfully disrupted. Three of these gene disruptions (*recJ*, *sbcD* and *recA*) required either reversing the polarity of the gentamicin resistance cassette or adding (or removing) a T7 transcriptional terminator. Previous attempts at *recA* insertional mutation have either been unsuccessful [41] or have resulted in a single clone [27]. In this study multiple *recA* disruptions were obtained only in the absence of a T7 terminator and with the *aacC1* in the reverse orientation relative to *recA*, underscoring the importance of the transcriptional features (direction of expression and the presence or absence of a transcriptional terminator) in the drug resistance cassette when attempting *B. burgdorferi* gene disruptions. It is also worthy of note that although our gene disruption mutants carry both an ∼500 bp deletion of the target gene as well as an insertion of the *aacC1* gene, expression of a partial gene product with some level of functionality cannot be rigorously ruled. Antiserum to the 17 disrupted genes is not currently available and immunoblotting experiments could therefore not be performed.

In addition to the 17 successful gene disruptions, we were unsuccessful in obtaining disruptions of *dnaB* (a replicative helicase), *recB* or *recC* (subunits of a recombinational helicase/nuclease) and *hbb* (an accessory factor with properties of *E. coli* HU and IHF, which introduces sharp bends in DNA [47],[48],[49]). Whether these genes are essential functions for *B. burgdorferi*, as is expected for *dnaB*, or whether our gene disruption conditions for these loci are still not ideal remains to be established. The primary purpose for the construction of mutants described here was to study the possible role of genes in question on recombinational switching at the *vlsE* locus. The effect of the 17 mutations on generalized recombination and DNA repair is currently under study and will be reported elsewhere.

Candidate genes involved in antigenic variation were identified by mouse infections in C3H/HeN and SCID mice. Initial screening for switching utilized an RFLP assay in wild-type mice followed by DNA sequencing of clones recovered from organ harvest cultures from SCID mice at 35 days post-infection. Analysis of the number of switching events and the source (silent cassette) of the new sequences at *vlsE* is often difficult to establish due to sequence redundancy in different cassettes and the complexity of the resulting switch genotypes (see [17]); hence, an exact method for determining the number of switches in all clones does not exist. Sequencing data was, therefore, analyzed by two independent methods. First, the number of clones that contained switches for each genotype was counted and compared to that of wild-type *B. burgdorferi* (**Fig. 3**). This approach provides information on the number of clones where recombinational switching occurs, but the number of switching events or extent of recombinational switching in each clone is not considered. The second method involved counting the number of nucleotide changes as an approximate indicator of the degree of switching (**Table S3**). Because switching events at *vlsE* usually involve short stretches of DNA [17], this method is expected to provide a reasonable estimate of the degree of switching. Although the binary alternative outcome for the first method results in a preferred statistical analysis, it is not currently known which of the two methods of estimating the extent of switching is more accurate. As discussed below, the data and conclusions from both methods of analysis were concordant.

Finally, it is noteworthy that other than the *ruvAB* mutants, which are required for switching at *vlsE*, the remainder of the genes we disrupted were dispensable for animal infection. The major DNA assault expected by a microbe upon animal infection is oxidative DNA damage originating from the innate immune response. However, due to a lack of iron in *B. burgdorferi*, oxidative damage of DNA appears to be much lower than in other organisms, such as *E. coli* [50]. Hence, DNA repair functions may be less important in protecting the pathogen from exogenous sources of DNA damage. Nonetheless, it is of interest that several of the recombination, repair and replication mutants examined in this study displayed altered infection phenotypes that were not attributable to an obvious deficit in *vlsE* switching in C3H/HeN mice (**Table 2**). Various levels of decreased infectivity were observed at days 7, 21 and 35 in comparison with the 100% infectivity displayed by all 18 wild-type control mice. Surprisingly, wild-type levels of infectivity for these mutants was restored in SCID C3H/HeN mice lacking an acquired immune response (**Table 3**). The mechanism for decreased infectivity in wild-type that is rescued in SCID mice remains open to speculation at this time.

### *recA* is not required for recombinational switching at *vlsE*

Homologous recombination in bacteria is typically initiated by RecA-mediated pairing and strand invasion [51],[52],[53]. It has been previously reported that a *recA* gene disruption in *B. burgdorferi* did not affect switching at *vlsE* [27]. Because the *recA* gene in *B. burgdorferi* is not easily disrupted [41] and because a single clone with the disrupted gene was used to assess the role of *recA* in recombinational switching at *vlsE* [27], we constructed several *recA* knockouts and tested two of them for switching at the *vlsE* locus. Our results confirm the previous findings that *recA* is not required [27]. This raises the interesting question of how pairing and strand invasion is initiated for recombinational switching at the *vlsE* locus. The lack of a requirement for the RecA protein, and the unidirectional segmental gene conversion events that characterize switching at the *vls* locus in *B. burgdorferi* argue for the need of a specialized protein(s) to help mediate the process. The expendability of RecA for antigenic variation in *B. burgdorferi* is also a stark difference from antigenic variation systems in other organisms, as will be discussed below.

### A role for the *ruvAB* encoded branch migrase in recombinational switching at *vlsE*

The RuvAB complex is required for homologous recombination and facilitates ATP-dependent branch migration of heteroduplex DNA in Holliday junctions [43],[44],[45]. RuvA tetramers bind and unfold the heteroduplex DNA and recruit RuvB hexamers, which function as a helicase to move DNA through the RuvAB complex. *B. burgdorferi* RuvA and RuvB share 32% and 48% identity with their *E. coli* orthologues, respectively. *ruvAB* mutants in *E. coli* have only modest defects in homologous recombination. However, these defects become significant when there are also mutations in other recombination proteins, such as *recBC*, *recG* and *sbcBC* [54],[55]. The role of RuvAB in DNA repair and recombination has not been previously investigated in *B. burgdorferi*.

The observed infection phenotype of both *ruvA* and both *ruvB* mutant strains reported here was as previously observed for strains lacking either lp28-1 [16],[17],[20],[21] or the *vls* locus [16],[17],[20],[21], where switching cannot occur. Infection of wild-type mice was 100% at day 7 with apparent complete clearance at day 21 (Table 2). Complete rescue of the persistence defect for *ruv* mutant strains was observed in all cases in SCID mice (Table 3). A difference in phenotype between strains lacking either lp28-1 or the *vls* locus, with those carrying a *ruv* mutation is that at 35 days post-infection spirochetes could be recovered in some organ harvest cultures from wild-type mice infected with the *ruv* mutants. DNA sequence analysis revealed that a single switch variant was present in spirochetes from a given tissue, or from both tissues in the two mice where positive cultures were recovered from two sites. These results are indicative of low frequency switching in the mutants, resulting in occasional survival and selection of a single switch variant. This phenotype has also been observed by [28].

The phenotype and the dramatic inhibition of switching at *vlsE* (**Fig. 3**) of the strains carrying the *ruvA* and *ruvB* gene disruptions in this study identify the first protein factors involved in switching and support a mechanism involving branch migration of a recombination intermediate for antigenic variation in *B. burgdorferi*. Although we were unable to complement our *ruv* mutations (data not shown), genetic complementation in *B. burgdorferi* is frequently difficult to achieve, for reasons not currently understood. We nonetheless argue for the absence of secondary mutations in the mutant *B. burgdorferi* strains based upon the following arguments: 1) Our studies were performed with two independent mutations in both the *ruvA* and *ruvB* genes. The four independent mutant strains demonstrated the same phenotype, making the existence of secondary mutations exceedingly remote. 2) Although not a strict genetic complementation, the rescue of persistent infectivity in all four *ruv* mutants following the infection of SCID mice is compelling evidence for the absence of any secondary mutations affecting infectivity. 3) Similar results and conclusions with *ruv* mutants made by transposon mutagenesis in the accompanying idependent study from the Norris lab [28] corroborate the findings presented here and our combined results provide compelling evidence for a RuvAB role in switching at *vlsE*.

A companion protein to RuvAB in most bacteria is the Holliday junction resolving enzyme RuvC. *B. burgdorferi* does not encode a RuvC orthologue and has no characterized junction resolving enzyme. This leaves unanswered the question of how recombination intermediates involved in homologous recombination or switching at *vlsE* are processed. A series of putative LE family exonucleases encoded by the cp32 family of circular plasmids has been proposed as possible substitutes for RuvC in *B. burgdorferi* [56]. The actual function of these λ exonuclease-type proteins remains to be established and simultaneous disruption of all of them (∼9) is outside the realm of possibility with current genetic methods available for *B. burgdorferi*.

### A possible role for *mutL* in recombinational switching at *vlsE*?

*mutL* and *recJ* are both players in bacterial mismatch repair [57],[58],[59]. In *E. coli* MutL acts as a liaison between MutS, which recognizes the mismatch, and MutH which is responsible for introducing a nick on either side of the mismatch. Both *mutS*1 and *mutS2* are present in the *B. burgdorferi* genome, but disruption of either gene did not affect the infectivity phenotype in wild-type mice or switching as assayed by RFLP. There is no identifiable *mutH* orthologue in *B. burgdorferi* [25]. Disruption of *mutL* resulted in a modest decrease in switching at *vlsE* that was significant (**Fig. 3**) or near significant (**Table S3**) in heart and bladder but not in ear and joint. The results did not allow a clear-cut conclusion on the involvement of MutL in switching as demonstrated for RuvA and RuvB. Further analyses will be required to derive an unambiguous answer to this question. It is possible that MutL plays a role in recombinational switching at *vlsE*, but that another *B. burgdorferi* protein can substitute for MutL because of functional redundancy. In such a case a double knockout will be required for further investigation; however, a functional paralogue of MutL has not been identified in *B. burgdorferi* at this time. It is also possible that the reduction in switching in *mutL* mutants results from a decreased level of fitness and a slower growth rate of the mutant in the mouse **(** **Table 2**).

RecJ is a 5′ to 3′ exonuclease that in *E. coli* can promote mismatch excision and prepares DNA for strand invasion by creating the single-stranded 3′-overhang [53]. Disruption of *recJ* resulted in a decrease in infectivity in wild-type mice at 21 days and a modest decrease in switching at *vlsE* that was significant only in the heart for both methods of analysis (**Fig. 3** and **Table S3**). Again, the results did not permit an unambiguous conclusion as to the possible involvement of RecJ in switching at *vlsE*. The slightly decreased *vlsE* switching phenotype observed in *B. burgdorferi* could be a result of redundancy of function for *recJ* and *recD* as has been previously reported in *E. coli* [60],[61]. A *recD recJ* double mutant, if viable, might provide further information regarding the role of *recJ* in *vlsE* recombination. Alternatively, the decrease in switching might simply reflect a decreased level of fitness and infectivity of the mutant spirochete in the mouse and there may be no direct role of RecJ in switching at *vlsE*.

Finally, it is noteworthy that *mutL* and *recJ* were the only genotypes sequenced that also contained non-templated nucleotide changes (NTCs). *mutL* had five NTCs while *recJ* had six for a total of eleven across 7 clones. The NTCs in this study occurred predominantly in invariable regions where nucleotide changes are not normally observed. An explanation for why NTCs were only observed in *mutL* and *recJ* could be due to their involvement in the mismatch repair pathway. These data suggest that these repair proteins are normally operative at *vlsE* to correct mismatches and would, therefore, normally be temporally and spatially positioned to play a role in the switching process as well. Recent work on switching at *vlsE* has reported that approximately 15% of wild-type *vlsE* variants carry NTCs [17]. This was not observed in our sequencing data with wild-type, *ruvA* or *ruvB* mutant clones. We have no explanation for this discrepancy.

### Comparison with other antigenic variation systems

While a wide variety of bacterial and protozoan pathogens employ antigenic variation systems driven by gene conversion [5],[6], the molecular details of the recombinational events underlying the process remain largely obscure. Information about some of the protein factors required for gene conversion events are available only from studies on the bacterial pathogen *N. gonorrhoeae* and the protozoan parasite *Trypanosoma brucei*. Both of these organisms require either RecA [37] or paralogues of its eukaryotic RecA counterpart, Rad51 [62],[63]. In contrast, both this study and a previous one [27] have shown that RecA is not necessary for switching at *vlsE* in *B. burgdorferi*. In *N. gonorrhoeae* the *recFOR* pathway is also involved and disruptions in *recQ*, *recO*, *recR* or *recJ* result in elimination or fairly dramatic reductions in antigenic variation [30],[32],[33]. In contrast, *B. burgdorferi* does not carry *recO*, *recR* or *recQ* orthologues and disruption of *recJ* did not demonstrate a clear role for its encoded protein in switching at *vlsE*. The Holliday junction resolution pathway (*ruvA*, *ruvB*, *ruvC and recG*) was also found to be important in *N. gonorrhoeae*, with disruptions in these genes resulting in a dramatic decrease in antigenic variation [31],[32]. In *B. burgdorferi* we found the RuvAB branch migrase to be required for switching at *vlsE*, however, there is no known *ruvC* orthologue and disruption of *recG*, which encodes a helicase that can function in Holliday junction migration, does not affect switching at *vlsE*. In summary, the process of recombinational switching at the *vlsE* locus shows very dramatic differences in protein requirements compared to the antigenic variation process in *N. gonorrhoeae*, with only the RuvAB branch migrase in common. Further studies on the recombinational switching underlying antigenic variation will be required to unravel the elusive molecular details of this fascinating process.

## Materials and Methods

### Bacterial strains and transformation

Infectious *Borrelia burgdorferi* 5A4, derived from the type strain B31 [40], was cultivated in BSK-II medium prepared in-house [64], supplemented with 6% rabbit serum (Cedarlane Laboratories, Burlington, ON, CA)) and incubated at 35°C (with a 1.5% CO_2_ environment for plating). Bacterial density was determined using a Petroff- Hausser Chamber (Hausser Scientific Partnership) and dark-field phase contrast microscopy with a Nikon Eclipse E400 microscope. *E. coli* DH5α was used for all knockout plasmid construction and maintenance. *B. burgdorferi* 5A4 were transformed as previously described with 25–50 µg of knockout plasmid DNA [65],[66]. Following transformation, the cell suspensions were immediately added to 10ml of pre-warmed BSK II supplemented with 6% rabbit serum. The transformations were allowed to recover for 24 hours at 35°C with 1.5% CO_2_. Recovery cultures were added to BSK II with 6% rabbit serum to a final volume of 50–100 ml and supplemented with 200 µg/ml gentamicin after which 250 µl aliquots were distributed into 96-well plates and incubated at 35°C and 1.5% CO_2_ until some wells with a visible color change from red to yellow were observed, usually between 8–12 days. Yellow wells were chosen for PCR analysis.

### Knockout plasmid construction

Primers for amplifying a centrally located ∼1500 bp portion in the gene of interest were designed according to the published sequence information (accession numbers NC_001318 and NC_001852) [25]. The target was amplified by PCR using Phusion High-Fidelity DNA Polymerase (Finnzymes), used for all subsequent PCRs unless otherwise noted using 80ng of genomic DNA template from *B. burgdorferi* B31 5A4 [40]. In early experiments the PCR product was cloned into the pJET1.2/blunt vector (Fermentas) and subsequently into the pCR BluntII-TOPO vector (Invitrogen). Plasmid DNA was isolated using the GeneJet Plasmid Miniprep Kit (Fermentas). Inverse PCR was employed to generate the knockout plasmid backbone [26]. Outward-oriented primers (see **Table S1**) with 5′ NheI restriction sites were designed within the target gene such that the amplicon produced did not contain approximately 500bp from the center of the target gene (see **Fig. S1**). The inverse PCR product was purified using the QIAquick PCR Purification Kit (Qiagen). The *flgB* promoter-driven gentamicin resistance cassette (*aacC1*) was used to disrupt the *B. burgdorferi* target gene. This cassette was incorporated into the knockout plasmid by amplifying the *flgB* promoter-driven gentamicin resistance gene from the plasmid shuttle vector pBSV2G [67] using primers B415 and B416 containing 5′ Nhe I sites. A gentamicin resistance cassette with a T7 transcriptional terminator (5′ CTG CTA ACA AAG CCC GAA AGG AAG CTG AGT TGG CTG CTG CCA CCG CTG AGC AAT AAC TAG CA TAA CCC CTT GGG GCC TCT AAA CGG GTC TTG AGG GGT TTT TTG 3′) was also used. This cassette was constructed using overlap extension PCR. The gentamicin resistance gene was amplified from pBSV2G using primers B820 and B1350, and the T7 terminator, originally from the pGEM-T easy vector (Promega), was amplified from a plasmid construct (pTAKanT7t) generously provided by Scott Samuels using primers B1349 and B1345. Following NheI (New England BioLabs) digestion the gentamicin resistance cassette and knockout plasmid backbone were ligated and used to transform DH5α, with selection using 10µg/ml gentamicin, with the addition of 50µg/ml kanamycin when the pCR BluntII-TOPO vector was used.

### Confirmation of *B. burgdorferi* gene disruption

Transformants were analyzed by PCR to identify clones with *bona fide* gene disruptions and to distinguish them from merodiploids. PCR was performed using Taq polymerase (New England BioLabs) and a combination of primers to confirm legitimate allelic exchange (see **Fig. 1**). Primers B348 and B349 were used to amplify the gentamicin resistance cassette, the knockout primers (KO/f and KO/r) for each mutant were used to confirm gene disruption. The target gene primers (target/f and target/r) for each mutant were used to confirm the correct insertion size upon recombination. Finally, primers B349 and B1281 were used in conjunction with the target primers to verify the correct insertion site and integrity of the recombination boundaries.

Southern hybridization analysis was used for verification of legitimate allelic exchange in the mutants selected for further study. Approximately 600ng of genomic DNA, prepared using the Wizard Genomic DNA Purification Kit (Promega) or mini-genomic DNA preps [68], was digested with HindIII (New England BioLabs) and separated on a 1.2% agarose gel run at 75V for 1.5 hours. After staining with 0.5µg/ml ethidium bromide to confirm complete enzymatic digestion the DNA was depurinated, denatured and neutralized as previously noted [69]. DNA was transferred to membranes (Hybond-N+ Amersham) and cross-linked using the UV Stratalinker 1800 (Stratagene). The *gent* probes were prepared from the PCR product of pBSV2G using primers B348 and B349. The KO/f and KO/r primers specific to each mutant were used to generate the probes using PCR from the genomic DNA of *B. burgdorferi* 5A4. The probes were labeled with [α-^32^P] dCTP by random primer labeling with a Random Primers DNA Labeling System Kit (Invitrogen). Standard procedures were used to pre-hybridize, hybridize and wash the blots [69], after which they were exposed to phosphor screens and analyzed with a Cyclone Phosphoimager (Packard). PCR analysis of plasmid content was performed as previously described to ensure the mutant clones contained the full plasmid complement required for infectivity [40],[70].

### Mouse infections

All animal infections were carried out in accordance with approved protocols from the University of Calgary Animal Research Centre and were approved by the University of Calgary Animal Care Committee. Three to four week old male C3H/HeN (wild-type) or three to five week old male C3H.C-*Prkdc^SCID^*/IcrSmnHsd (SCID) mice (Harlan, Indianapolis, IN) were inoculated with 200 µl of 1×10^4^ spirochetes/ml, in two 100 µl doses via dorsal subcutaneous and intraperitoneal injection. At seven days post-infection, 50 µl of blood was taken from the saphenous vein on the hind leg of the mouse under aseptic conditions. The exposed vein was opened using a needle prick and the pooled blood was drawn with a pipette. The pipette tip used to draw the blood was first coated with 0.5M EDTA to prevent clotting. The blood sample was suspended in 1.7ml BSK II supplemented with 6% rabbit serum and 1× *Borrelia* antibiotic cocktail (20 µg/ml phosphomycin, 50 µg/ml rifampicin and 2.5 µg/ml amphotericin B) and cultivated as described above. Ear biopsies were performed at days 14 and 21 and the recovered material was cultivated in 1.5ml of BSK II supplemented with 6% rabbit serum and 1× *B. burgdorferi* antibiotic cocktail for one to five weeks. The presence or absence of spirochetes was periodically monitored by dark-field microscopy. When necessary, a 35 day organ harvest was performed and the heart, bladder, joint and ear biopsy samples were removed aseptically and cultured in 1.7ml of BSK II supplemented with 6% rabbit serum as noted above.

### *vlsE* switching assay

Switching at the *vlsE* locus was determined by a restriction fragment length polymorphism (RFLP) assay using the 775 bp product of PCR amplification of the *vlsE* expression site using primers B248 and B249 [16]. This crude switching assay was performed on week three ear biopsy cultures when available, or on week 5 organ harvest cultures. Phusion polymerase (Finnzymes) was used for PCR and the product was purified using the QIAquick PCR Purification Kit (Qiagen). Approximately 200ng of PCR product was digested with 2 units of HphI (New England BioLabs) for 1.5 hours. Reaction products were analyzed on a 1.2% agarose gel run at 75 V for 1.5 hours in Tris-acetate buffer, stained with 0.5 µg/ml ethidium bromide and images acquired using a FluorChem 8900 imaging system.

### *vlsE* cloning and sequencing

For detailed analysis of switching at *vlsE*, mutant *B. burgdorferi* strains were used to infect C3H/HeN SCID mice. Switching in SCID mice was characterized through sequencing of the variable regions of the *vlsE* expression site. PCR amplification was performed using primers B248 and B249 on 1µl of BSK-II cultures, grown to a density of 1×10^6^ spirochetes/ml, taken from glycerol stocks of the heart, bladder, joint and ear organ harvests for each mouse. Reaction conditions were as follows: 98°C for 2 minutes, 28 cycles of 98°C for 10 seconds and 72°C for 30 seconds, followed by a final extension of 72°C for 5 minutes. PCR products were visualized and quantified on a 1% agarose gel run in TAE buffer at 75V for 1.5 hours, stained with 0.5 µg/ml ethidium bromide. Equimolar portions of the PCR product from each of the four mice (two mice for each clone) and each tissue type were combined to give a total of four samples: heart, bladder, joint and ear for each genotype investigated. These PCR products were run on a 1% agarose gel and the 775 bp PCR product was excised and gel purified using the Qiagen Gel Extraction Kit (Quiagen). The PCR fragments were cloned into the pJET1.2/blunt vector (CloneJet, Fermentas) and used to transform *E.coli* DH5α. The transformations were plated on LB agar plates containing 100 µg/ml carbenicillin at 37°C. In preparation for sequencing, 10 colonies of each tissue type for each mutant were picked and grown in five ml LB supplemented with100 µg/ml carbenicillin for a total of 40 samples from each genotype. These cultures were grown overnight at 37°C and plasmid DNA was isolated using the Qiagen 96 Turbo miniprep kit (Qiagen). The University of Calgary Core DNA Services sequenced 500 ng of the plasmid DNA with the pJET1.2forward sequencing primer (CloneJet, Fermentas) in a 96 well format using an Applied Biosystems 3730XL 96 Capillary Sequencer (http://www.ucalgary.ca/dnalab/).

### Analysis of sequencing results

Alignments comparing the cloned *vlsE* sequencing results for each tissue type in each mutant to the parental *vlsE* sequence of *B.burgdorferi* 5A4 were performed using the Seqman DNASTAR-Lasergene v6 Software. Templated nucleotide changes, those corresponding to the sequence of at least one silent casette, were counted in each variable region as well as the invariable regions and noted (**Table S3**). Additionally, non-templated changes were documented (data not shown). It is important to note that each of the 10 sequences obtained for each tissue type for each mutant were different and, therefore, all sequenced clones represented completely independent switching outcomes. Results were analyzed on a tissue-specific basis via two methods (**Fig. 3**). The first method took into account how many clones from each tissue type switched and how many retained the parental *vlsE* sequence. The two-tailed Fisher's Exact test was used to determine the P-values of the mutant switch events (GraphPad Prism). The second method used to analyze switching was a comparison of data based on the number of nucleotide changes in each tissue for each mutant (**Table S3**). A two-tailed non-parametric Mann-Whitney student t-test was used to determine the P-values of these data (Graph Pad Prism).

## Supporting Information

Figure S1 Strategy for the construction of knockout plasmids. Construction of the knockout plasmids was accomplished by PCR amplification of an approximate 1.5 kb central portion of the target gene (yellow) inserted into a commercial blunt-end cloning vector. Inverse primers (see Materials and Methods and Table S1) with NheI sites were used to amplify the vector and target gene minus a central ∼0.5 kb portion of the target gene. Following digestion with Nhe I the product of the inverse PCR reaction was ligated to a gentamicin resistance cassette under the control of the *flgB* promoter (blue) with Nhe I sticky ends. The knockout plasmids were propagated in *E. coli* strain DH5α.
(0.24 MB PDF)

Figure S2 Gene disruption was demonstrated by PCR analysis (see Fig. 1B) and subsequently confirmed by Southern hybridization of genomic DNA. In the Southern blot shown, DNA from *mutL*, *sbcD* and *ruvA* disruptions was digested with HindIII and run on a 1.2% agarose gel with a 1kb molecular weight ladder (M). Probes complementary to the gentamicin resistance cassette were used to probe for the gent insertion (left panel). pBSV2G served as the positive control (c+) and *B. burgdorferi* 5A4 genomic DNA served as the negative control (wt). *mutL*1 and 2, *sbcD*1 and 2 and *ruvA*1 and 2 clones displayed the expected fragments of 5.5kb, 7.4kb, and 2.8kb respectively. In the right hand panel, probes complementary to the deleted portion of the target gene were generated using the knockout primers (see Table S1). The expected size of the hybridization fragments for these blots was determined based upon the nearest flanking HindIII sites to targeted gene in the *B. burgdorferi* B31 genomic DNA sequence [25]. *mutL^+^*, *sbcD^+^* and *ruvA+* wild type genomic DNA provided the expected signals of 4.8kb, 6.4kb and 1.7kb respectively. As expected, no signals were observed for the *mutL*, *sbcD* and *ruvA* knockout genotypes indicating the central portion of the target genes was replaced. Equal amounts of DNA were loaded in each lane.
(1.23 MB PDF)

Table S1 Primers used in this study.
(0.03 MB PDF)

Table S2 Southern blot analysis.
(0.02 MB PDF)

Table S3 Expanded C3H/HeN SCID *vlsE* aequence data.
(0.14 MB PDF)
