# Supplementary figures and images for: Investigation of the Genes Involved in Antigenic Switching at the vlsE Locus in Borrelia burgdorferi: An Essential Role for the RuvAB Branch Migrase
Source: PLoS Pathog. 2009 Dec 4;5(12):e1000680. doi: 10.1371/journal.ppat.1000680 (PMC2779866; doi:10.1371/journal.ppat.1000680)

## Supplementary Fig. 1

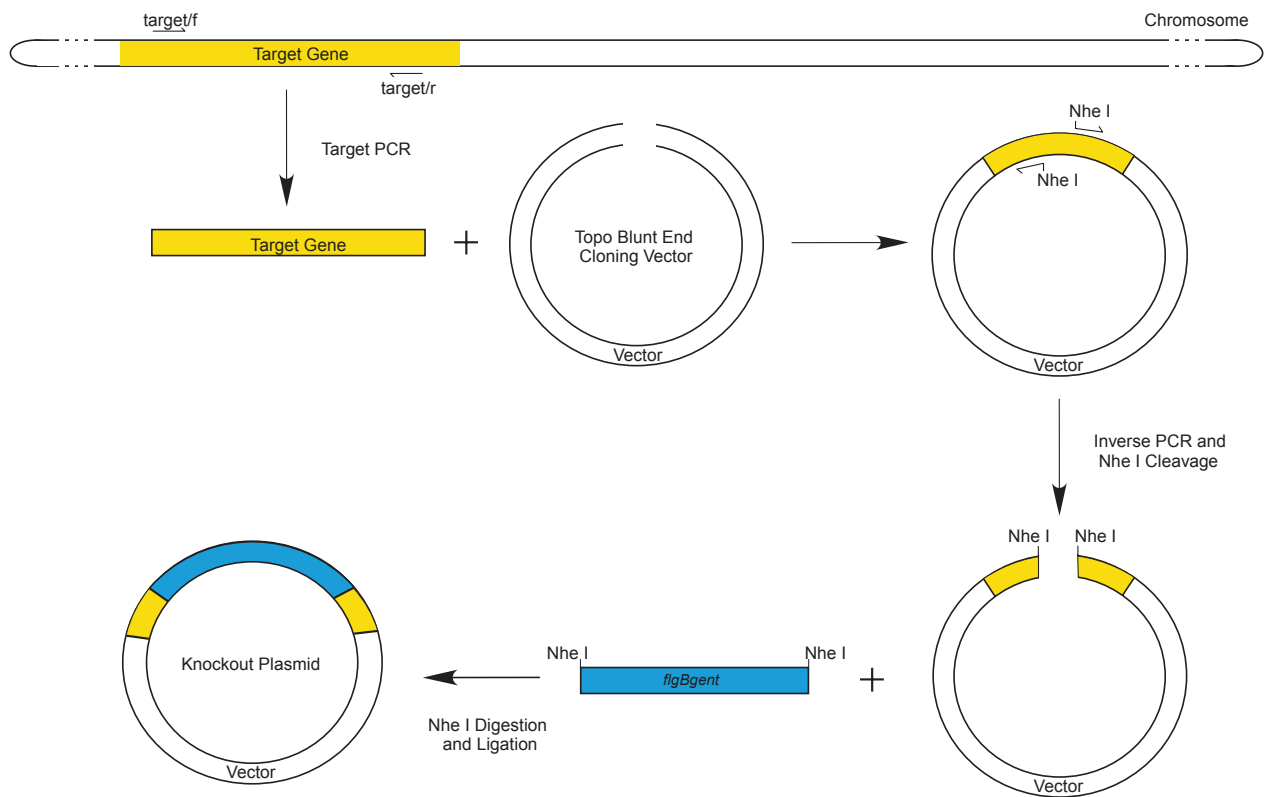

Supplement: Figure S1 — Strategy for the construction of knockout plasmids. Construction of the knockout plasmids was accomplished by PCR amplification of an approximate 1.5 kb central portion of the target gene (yellow) inserted into a commercial blunt-end cloning vector. Inverse primers (see Materials and Methods and Table S1) with NheI sites were used to amplify the vector and target gene minus a central ∼0.5 kb portion of the target gene. Following digestion with Nhe I the product of the inverse PCR reaction was ligated to a gentamicin resistance cassette under the control of the flgB promoter (blue) with Nhe I sticky ends. The knockout plasmids were propagated in E. coli strain DH5α. (0.24 MB PDF) [file ppat.1000680.s001.pdf]

Supplementary Fig. 2

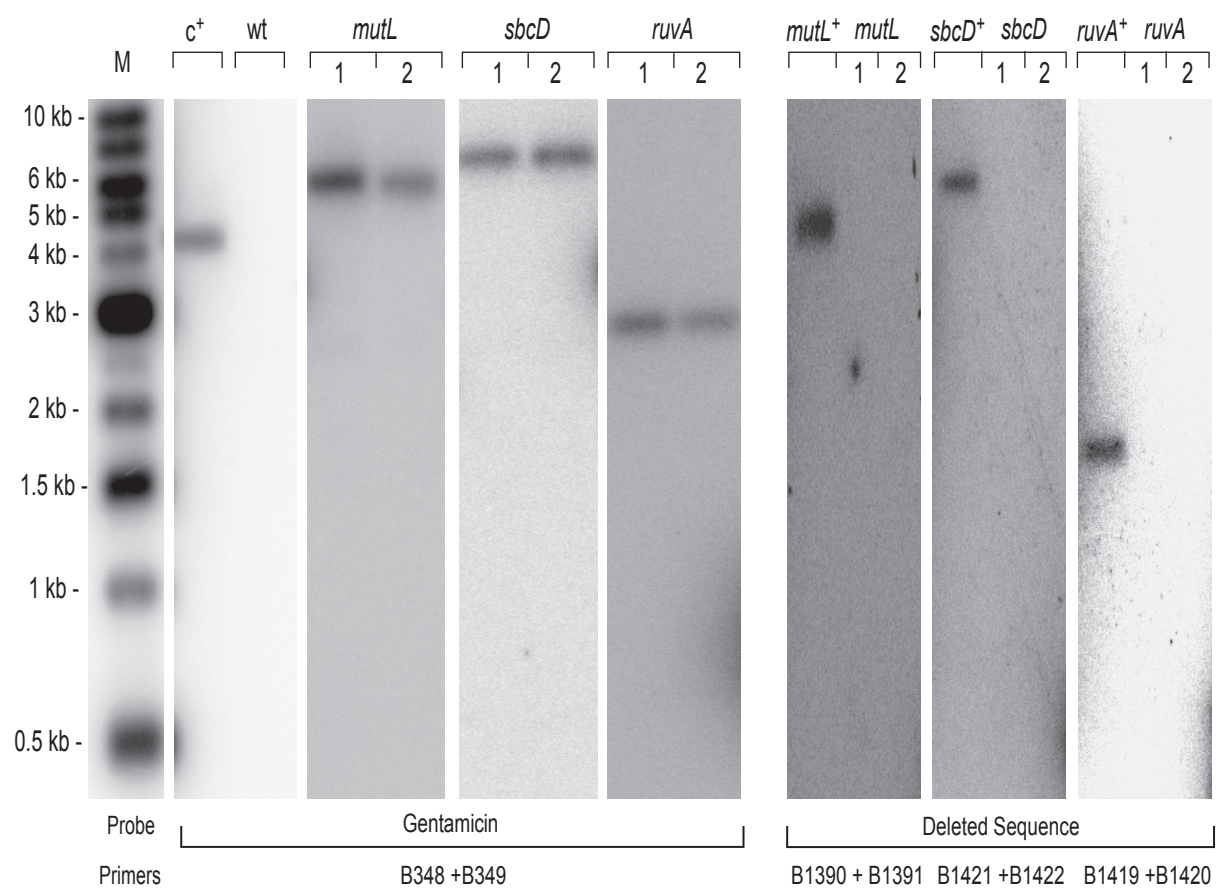

Supplement: Figure S2 — Gene disruption was demonstrated by PCR analysis (see Fig. 1B) and subsequently confirmed by Southern hybridization of genomic DNA. In the Southern blot shown, DNA from mutL, sbcD and ruvA disruptions was digested with HindIII and run on a 1.2% agarose gel with a 1kb molecular weight ladder (M). Probes complementary to the gentamicin resistance cassette were used to probe for the gent insertion (left panel). pBSV2G served as the positive control (c+) and B. burgdorferi 5A4 genomic DNA served as the negative control (wt). mutL1 and 2, sbcD1 and 2 and ruvA1 and 2 clones displayed the expected fragments of 5.5kb, 7.4kb, and 2.8kb respectively. In the right hand panel, probes complementary to the deleted portion of the target gene were generated using the knockout primers (see Table S1). The expected size of the hybridization fragments for these blots was determined based upon the nearest flanking HindIII sites to targeted gene in the B. burgdorferi B31 genomic DNA sequence [25]. mutL+, sbcD+ and ruvA+ wild type genomic DNA provided the expected signals of 4.8kb, 6.4kb and 1.7kb respectively. As expected, no signals were observed for the mutL, sbcD and ruvA knockout genotypes indicating the central portion of the target genes was replaced. Equal amounts of DNA were loaded in each lane. (1.23 MB PDF) [file ppat.1000680.s002.pdf]
