# Supplementary material for: Investigation of the Genes Involved in Antigenic Switching at the vlsE Locus in Borrelia burgdorferi: An Essential Role for the RuvAB Branch Migrase
Source: PLoS Pathog. 2009 Dec 4;5(12):e1000680. doi: 10.1371/journal.ppat.1000680 (PMC2779866; doi:10.1371/journal.ppat.1000680)
Supplement: Table S1 — Primers used in this study. (0.03 MB PDF) [file ppat.1000680.s003.pdf]

Supplementary Table 1. Primers used in this study

Primers used for knockout plasmid construction

Genotype	Target (f/r)	Inverse	Knockout (KOf/r)
priA	B1183f-ACAGAACATAACATTGATTTAGC	B1235-ATGCAATCCGCTAGCTCGTCCATAATTATTAATTTCA	B1282-CACGAAACTACATACAAATCTGA
	B1184r-CAACATTTTTGTTTAGCAGA	B1236-ATGCAATCCGCTAGCATGCGAATCAAAAGATATTAAA	B1283-GCAATGGCTTGCTGTTTTTG
recB	B1185f-TTTTGCTATTTCTAGTAATTTGC	B1452-TCTTTGGTGATATTTGCA	B1284-GCAATTTAGCTCTTGTTGCTC
	B1186r-ACTATTAAATCAACAAACACAATAG	B1453-AACAATAAAATATTTGCTTGC	B1285-CCTATGAAACAACACTTGAGATC
recG	B1187f-CCTTTCGAAATTCTGCTT	B1239-ATGCAATCCGCTAGCTATCTCATCAATAGAAATTTTTTG	B1286-TCTAAGCCAATGAATAGATTGCT
	B1188r-TTAATCGAAATTTGCCAG	B1240-ATGCAATCCGCTAGCGTTTTGCATTAACACTTTTTG	B1287-GGTGTTGCAGACATTAAAAGC
mag	B1223f-CGCCGTTACAAGATAATTT	B1303-CATTTCCTAGCTAGCAAGAGGTTTGAATCTAGATTTTA	B1388-TGTAAAAAAAGCTCATTATTCC
	B1224r-AGCTATGATCCCTTAAGCATAT	B1304-CATTTCCTAGCTAGCATACACATAAGAATATCCTCCTATT	B1389-ATAATATATGGTATGCATTATATGTTT
rep	B1299f-TCCTCTTAGTAATCTTATGGTTT	B1309-CATTTCCTAGCTAGCGGCAGGAATATTTGAAA	B1394-GGGGCTAATTATAACAACAT
	B1300r-CTTCAACAGCAATAAAAAATAC	B1310-CATTTCCTAGCTAGCTTTTAACAAAGTTAATGCTAGTTC	B1395-CCCTTCTTGGAACATTAAT
BBG32	B1369f-CTGTTTGTAGCTTTTCTAAGAA	B1386-CATTTCCTAGCTAGCATTACTACAAAATTTGATTCCC	B1429-GTTGGTAAAACGGCTTTT
	B1370r-CCTTTTGTAAATTCTTTTCTACA	B1387-CATTTCCTAGCTAGCCTATAAAATTTTCGAATTGAAA	B1430-TAAGCGTCCAGTTCACTT
dnaB	B1361f-CTAATACTGGTGGTCTTGTT	B1378-CATTTCCTAGCTAGCGCATTTTATCTGGACAAGA	B1423-TATTTTGAACTTTAAAGCTATCA
	B1362r-GGCTTTAGCAGCTTTTTC	B1379-CATTTCCTAGCTAGCAGCATGATAGAGGTTCTTACTAG	B1424-AAAGTTATTGCAGAACGTG
recJ	B1215f-GATTTTGGACTTAATGTGAGTT	B1225-ATGCAATCCGCTAGCAGTTTGTTTGAGTTAATTAGGG	B1288-GCATAAACATAAACACAATTGATATTAGTG
	B1216r-TTGGAAGAATAGCATCTATTG	B1226-ATGCAATCCGCTAGCGCTTGAAAAAGCAGATATTG	B1289-CTTCCCGTTGAGTTTAGTATTGG
nucA	B1301f-TCGATTTTACGAAAAATTG	B1311-CATTTCCTAGCTAGCTTTTAACAAAGTTAATGCTAGTTC	B1396-TTAAAAGAAGTACCAAATTCTTT
	B1302r-GGCAAAGATCAAAATAATGT	B1312-CATTTCCTAGCTAGCAAAAAACACGCATTCTTG	B1397-AATTTTTGAATCAAGTTTTTC
recC	B1365f-AATTGGGCTTTCTAGTTTAA	B1382-CATTTCCTAGCTAGCGCTTGAAGAAAGTCTTGAA	B1425-GATAAAATTCAATTTTTATTTCAT
	B1366r-AAACTATGAATCTACTCTTGTTAAAG	B1383-CATTTCCTAGCTAGCTTGTTGTTACTTAGTAGGTCAAA	B1426-TGAAAAAAATTTAATATATCAACATC
mutS1	B1067f-GGGATCTTGAGAAATACTCTCCTAAAG	B1069-GCGCTAGCTAGCTAGGGCGCATATAGGCTCTTTTGT	B1140-CCAGGCTAGATCATGTTGAATTTT
	B1068r-CAAGATCGTTGCCCTGCTTTTC	B1070-GCGCTAGCTAGCTAGGTCTCTTTCCCATTGCAGTTTTGC	B1141-TAGTGCTCAACAACAGGATGCC
mutS2	B1217f-CTAACCTTGTCTCCTATTTTAAA	B1227-ATGCAATCCGCTAGCGATGGATTTAGAGACAATGC	B1290-CGCATAGAAGCATTAACAACA
	B1218r-AAAAAATTTGCTTAATGAATTG	B1228-ATGCAATCCGCTAGCTTAATACATTTGAAATCTCGG	B1291-CACACCATCCTTTGTTAAAGG
mutL	B1295f-TTAATAATAGATAATGGAAGCG	B1305-CATTTCCTAGCTAGCATTTTCTATTACATTTCCATATACA	B1390-AATAAATTTAGGGTCTTAAAAAAA
	B1296r-AATAGTTGCGTATAAGTCAGATT	B1306-CATTTCCTAGCTAGCACACAAAGCAATATTGACC	B1391-TTTTGTTTGGCTCGTTTA
mfd	B1297f-GCATAAGGGGTTAATCTTT	B1307-CATTTCCTAGCTAGCTTTAAAAATTTTCCAATCAAA	B1392-TCTTTTTTTAAATGTATTGAAATG
	B1298r-AGAAGCCGGTAAAAATATAA	B1308-CATTTCCTAGCTAGCTTATTCTCTTTATTTGCCTAAAT	B1393-AAACAAGCTCTCTTGAAAAG
sbcC	B1221f-ATTGATCGATTAGAAATTGATC	B1231-ATGCAATCCGCTAGCAAATGCTTTTAATCTCATCA	B1292-TTGTTCCAAACTTTTAATGATGAGTTG
	B1222r-TTTGCAAAAAAGACAGACTAT	B1232-ATGCAATCCGCTAGCAGATTGATTGGAAAAAATTT	B1293-TCAAACTCCAAAGCATTAACCTC
sbcD	B1367f-GTGAGCAATTATAAAATTCTG	B1384-CATTTCCTAGCTAGCTTTCTCTGTAGGCATTTTC	B1421-TTTTTTAGAAAACAAGTATAAAGG
	B1368r-TTATTTATCCTCATATTCACCC	B1385-CATTTCCTAGCTAGCTGCATATTCATTTAATGAGA	B1422-TAGGAGATCCTGAATAAACAA
ruvA	B1359f-ATAATCTTCCATGGCAAT	B1376-CATTTCCTAGCTAGCCTTGAATCCACTGGTCTT	B1419-CTCGCTATTAATCAAAAGC
	B1360r-TCCATGTATAGTCTTTATGTGA	B1377-CATTTCCTAGCTAGCATAACTCAACTTTGTCTGACAA	B1420-AAATGAATTAAAACTTTTTGG
ruvB	B1200f-CGAAAATAGTATAAGCTTTTTAAGCTC	B1202-GCGCTAGCTAGCTAGGCTCAAGAGGGACTCCTCGT	B1249-CATAGGCTCAGACCAATAATAGAAG
	B1201r-CTTTGGTTTTCATTAAGACCATCC	B1203-GCGCTAGCTAGCTAGCACTCTTCTCATCAAGACCTGTT	B1250-TTTCTCTTTATTATCTCAACAAGCTC
recA	B1400f-TCGATCAATCCCCTAATA	B1402-CATTTCCTAGCTAGCTTGTAAAGAACAAGGTTGC	B1431-TCTTAACTCTAATCTTATTGCC
	B1401r-CAGGCAAAGATGAATCTTA	B1403-CATTTCCTAGCTAGCACCTTCTTTTTGCACCTC	B1432-GGGATAGCTGCTTTTATT
hbbU	B1444f-CAACAACTTTAACCACTTTTT	B1446-CATTTCCTAGCTAGCATCACGTTGCGTATTTTC	B1448-GATCTAGGACCTTAACATACTCC
	B1445r-TGTTATTTTATGAGTTTAGGAAAG	B1447-CATTTCCTAGCTAGCTATTCAAAGATATTTGATCAACA	B1449-TTAAAAATAATAATCTGAAATTAGAAA
nth	B1486f-GGCCAAATTTTCTCCTAAAAATT	B1488-CTAGCTAGCTAGCCCGCAATTATTGTAGATACTCAT	B1501-CTCCATACCTTTTTGAAAGGTACG
	B1487r-GCATTGATTCATGGATGGATT	B1489-CTAGCTAGCTAGCCAAATTATCTGTTGTTCTTGCA	B1502-GATCCAAGAATAACATTTGCCG

Primers used for plasmid content screen[a]

Plasmid	Forward	Reverse
lp21	B421-TGTGGTTGCTAAAACCCAAGCGT	B422-TTGTTTCTAATTGCTCTGAATTGCATCC
lp28-2	B423-CCCTCATCAAGTTTTTCCATGTGTTTTT	B424-AGGTGGCCTTTCCGAGCTTGTACCTTAC
lp28-3	B425-AACACTATCTTAAATGTCCCCCACAA	B426-GTGGAAGAGTGGTTATGGTCAATTTT
lp25	B427-AGAATTATGTCGGTGGCGTTGT	B428-ATTAAAGCCGCCTTTTCCTTGGT
lp28-1	B429-CGGGGATCCAGCCAAGTTGCTGATAAGGACGACCC	B430-ACGGCAGTTCCAACAGAACCTGTACTATCT
lp56	B431-ACTATTAAGACGAGCAATAAAAAGTCCA	B432-GACGAAGCAAAGAAGGATTTGGATCACC
cp26	B433-ATAGCCCATTCCAGACATTAAACCGCCT	B434-AGTTCCCCAAATAACAGCAATCTGCGA
lp17	B435-ACTGCAATCTGCCCAAGCTACATAATCT	B436-AAGGTAAGGACGGTTGTCTACATGGATT
cp32-9	B437-TATCAAAAAAGTGCTGTTTTATAG	B438-TAATCTCAAATATTCTTCTTTATG
lp38	B439-AGCAGGCAGAACAAAACATGCAAAAACTG	B440-TCCAAGCTATTTCTACGGCCTCTTTAGC
cp32-3	B441-GCAAGTTCCCACGATAACACACCCGTAT	B442-TTTTCATATCCCCTCCTAGCTTTATTGCC
lp36	B443-TTCTTATCCCTGACTTTCACTTTTGAGG	B444-TCCTTTACTTCTATGTTTTTACTTTCCTTGGT
cp9	B445-GGACTGGTATTTACTCCGGCTGATAGAGC	B446-CCTTAATGATGAGGCCGATGAAGTTGC
cp32-8	B447-GAAGATTTAAACAAAAAAATTGCG	B448-GTAATCACTTCTTTTTTACCATCG
lp54	B449-GCAAAATGTTAGCAGCCTTGACGAGAAA	B450-TAGATCGTACTTGCCGTCTTTGTTTTTT
cp32-1	B451-ACGATAGGGTAATATCAAAAAAGG	B452-AGTTCATCTAATAAAAATCCCGTG
cp32-2/7	B453-GGAATGTATTAATTGATAATTCAG	B454-GCGAACTAAATAGTGCCTTATGGG
cp32-6	B455-GACTTTACATAGTATAAATGCTTTTGG	B456-TCTCGTTTTATAAAATAAGTAGG
lp28-4	B457-TCACCTCAGCTAATCTATTTATCGACAC	B458-AAGCGCGGAGTTTTCGGCTG

Primers used for gentamycin resistance cassette cloning and transformant screens and sequencing
B348-CGCAGCAGCAACGATGTTAC
B349-CTTGCACGTAGATCACATAAGC
B1281-ATCGTCTATGCTTAAGCTCTT
B415-CATTTCCTAGCTAGCGGCGAATGGCGCGGCCGCCCTAGG
B416-CATTTCCTAGCTAGCACGCGTAAGCCGATCTCGGCTTG
B820-CATTTCCTAGCTAGCCCTAGGTAATACCCGAGCTTCAA
B1345-CATTTCGCTAGCCAGATCCGGATATAGTTCCTCCTTTC
B1349-CCCAAGTACCGCCACCTAATCAGAATTGGTTAATTGGTTGTAACACT
B1350-AGTGTTACAACCAATTAACCAATTCTGATTAGGTGGCGGTACTTGGG
pJET1.2forward-GGACTCACTATAGGGAGAGCGGC

[a]Primers used for plasmid content screen first reported in Purser JE, Norris SJ (2000). PNAS 97: 13865-13870.
