# Supplementary material for: Investigation of the Genes Involved in Antigenic Switching at the vlsE Locus in Borrelia burgdorferi: An Essential Role for the RuvAB Branch Migrase
Source: PLoS Pathog. 2009 Dec 4;5(12):e1000680. doi: 10.1371/journal.ppat.1000680 (PMC2779866; doi:10.1371/journal.ppat.1000680)
Supplement: Table S2 — Southern blot analysis. (0.02 MB PDF) [file ppat.1000680.s004.pdf]

Supplementary Table 2. Southern blot analysis

Gene	Locus	*gent* probe fragment (bp)	Gene disruption probe fragment (bp)
mutS2	*BB0098*	3000[a]	2918
recA	*BB0131*	2580	1628
recG	*BB0581*	3031	2322
rep	*BB0607*	2850	1168
nucA	*BB0411*	3762	3132
mag	*BB0422*	1340	395
mfd	*BB0623*	2023	1326
mutS1	*BB0797*	1409	725,459,185
recJ	*BB0254*	1454	400
ruvB	*BB0022*	3025	2313
ruvA	*BB0023*	2840	1681
sbcD	*BB0829*	7395	6458
sbcC	*BB0830*	1333	456
putative helicase	*BBG32*	4208	3740
priA	*BB0014*	5126	4434
mutL	*BB0211*	5514	4854
nth	*BB0745*	5303	4530

[a]Expected fragment is1763bp, the size difference is due to a deletion in an expected Hind III site
