# Supplementary material for: Investigation of the Genes Involved in Antigenic Switching at the vlsE Locus in Borrelia burgdorferi: An Essential Role for the RuvAB Branch Migrase
Source: PLoS Pathog. 2009 Dec 4;5(12):e1000680. doi: 10.1371/journal.ppat.1000680 (PMC2779866; doi:10.1371/journal.ppat.1000680)
Supplement: Table S3 — Expanded C3H/HeN SCID vlsE aequence data. (0.14 MB PDF) [file ppat.1000680.s005.pdf]

Supplementary Table 3. Expanded C3H/HeN SCID *vlsE* sequence data

Heart[a] — Templated nucleotide changes, variable regions

Genotype	Clone	1	2	3	4	5	6	C[b]	Total	P-value[c]
wt	1	0	0	0	1	0	0	0	1	
	2	0	2	1	7	0	4	0	14	
	3	0	1	0	0	0	0	0	1	
	4	1	0	0	0	0	0	0	1	
	5	1	0	1	1	0	0	1	4	
	6	0	0	0	1	0	7	0	8	
	7	0	0	1	0	0	0	0	1	
	8	0	0	0	2	1	0	0	3	
	9	1	0	0	3	5	4	0	13	
	10	0	0	0	1	0	0	0	1	
	**Total**	3	3	3	16	6	15	1	**47**	**n/a**
ruvB	1	0	0	0	0	0	0	0	0	
	2	0	0	0	0	0	0	0	0	
	3	0	0	0	0	0	0	0	0	
	4	0	0	0	0	0	0	0	0	
	5	0	0	0	0	0	0	0	0	
	6	0	0	0	0	0	0	0	0	
	7	0	0	0	0	0	0	0	0	
	8	0	0	0	0	0	0	0	0	
	9	0	0	0	0	0	0	0	0	
	10	0	0	0	0	0	0	0	0	
	**Total**	0	0	0	0	0	0	0	**0**	**<0.0002**
ruvA	1	0	0	0	0	0	0	0	0	
	2	0	0	0	0	0	0	0	0	
	3	0	0	0	0	0	0	0	0	
	4	0	0	0	0	0	0	0	0	
	5	0	0	0	0	0	0	0	0	
	6	0	0	0	0	0	0	0	0	
	7	0	0	0	0	0	0	0	0	
	8	0	0	0	0	0	0	0	0	
	9	0	0	0	0	0	0	0	0	
	10	0	0	0	0	0	0	0	0	
	**Total**	0	0	0	0	0	0	0	**0**	**<0.0002**
mutL	1	0	0	0	0	1	5	0	6	
	2	0	0	0	0	0	0	0	0	
	3	0	0	0	0	0	0	0	0	
	4	0	0	0	0	0	0	0	0	
	5	0	0	0	0	0	0	0	0	
	6	0	0	0	0	0	0	0	0	
	7	0	0	0	0	0	0	0	0	
	8	10	4	2	4	6	6	1	33	
	9	0	0	0	0	0	0	0	0	
	10	3	3	0	1	0	0	3	10	
	**Total**	13	7	2	5	7	11	4	**49**	**0.0565**
recJ	1	0	0	0	0	0	0	0	0	
	2	0	0	0	0	0	0	0	0	
	3	0	1	1	1	0	10	0	13	
	4	0	0	0	0	0	0	0	0	
	5	0	0	0	0	0	0	0	0	
	6	0	0	0	0	0	0	0	0	
	7	0	0	0	0	0	0	0	0	
	8	0	0	0	0	0	0	0	0	
	9	0	4	7	0	0	0	0	11	
	10	0	0	0	0	0	0	0	0	
	**Total**	0	5	8	1	0	10	0	**24**	**0.0093**

Bladder — Templated nucleotide changes, variable regions

Genotype	Clone	1	2	3	4	5	6	C	Total	P-value
wt	1	0	0	0	1	2	5	3	11	
	2	0	0	3	2	0	4	1	10	
	3	0	1	0	4	5	0	0	10	
	4	0	0	0	1	0	2	3	6	
	5	0	0	0	7	0	0	1	8	
	6	0	0	0	5	2	5	4	16	
	7	0	0	0	1	7	0	0	8	
	8	7	0	0	2	1	5	0	15	
	9	1	2	5	5	12	1	0	26	
	10	2	0	0	1	8	2	0	13	
	**Total**	10	3	8	29	37	24	12	**123**	**n/a**
ruvB	1	0	0	0	0	0	0	0	0	
	2	0	0	0	0	0	0	0	0	
	3	0	0	0	0	0	0	0	0	
	4	0	0	0	0	0	0	0	0	
	5	0	0	0	0	0	0	0	0	
	6	0	0	0	0	0	0	0	0	
	7	0	0	0	0	0	0	0	0	
	8	0	0	0	0	0	0	0	0	
	9	0	0	0	0	0	0	0	0	
	10	0	0	0	0	0	0	0	0	
	**Total**	0	0	0	0	0	0	0	**0**	**<0.0001**
ruvA	1	0	0	0	0	0	0	0	0	
	2	0	0	0	0	0	0	0	0	
	3	0	0	0	0	0	0	0	0	
	4	0	0	0	0	0	0	0	0	
	5	0	0	0	0	0	0	0	0	
	6	0	0	0	0	0	0	0	0	
	7	0	0	0	0	0	0	0	0	
	8	0	0	0	0	0	0	0	0	
	9	0	0	0	0	0	0	0	0	
	10	0	0	0	0	0	0	0	0	
	**Total**	0	0	0	0	0	0	0	**0**	**<0.0001**
mutL	1	0	0	0	0	0	0	0	0	
	2	9	0	2	4	4	2	1	22	
	3	0	0	0	0	0	0	0	0	
	4	0	0	0	0	0	0	0	0	
	5	0	0	0	0	1	5	0	6	
	6	0	0	0	0	0	0	0	0	
	7	0	0	0	0	0	0	0	0	
	8	0	0	0	0	0	0	0	0	
	9	0	0	0	0	0	0	0	0	
	10	0	0	0	0	0	0	0	0	
	**Total**	9	0	2	4	5	7	1	**28**	**0.0018**
recJ	1	0	0	0	0	1	5	0	6	
	2	10	5	2	11	0	0	0	28	
	3	0	0	0	0	1	0	0	1	
	4	0	0	0	0	0	0	0	0	
	5	1	0	1	1	0	6	0	9	
	6	0	0	5	18	0	0	0	23	
	7	4	0	0	3	0	2	0	9	
	8	3	11	2	0	0	6	0	22	
	9	0	0	1	1	0	6	0	8	
	10	0	0	0	0	0	0	0	0	
	**Total**	18	16	11	34	2	25	0	**106**	**0.3243**

Joint — Templated nucleotide changes, variable regions

Genotype	Clone	1	2	3	4	5	6	C	Total	P-value
wt	1	0	0	0	3	1	1	0	5	
	2	0	0	0	0	0	0	0	0	
	3	0	0	1	0	0	0	0	1	
	4	0	0	0	0	0	0	0	0	
	5	0	0	0	0	4	2	2	8	
	6	0	0	0	0	0	0	0	0	
	7	0	0	0	0	0	0	0	0	
	8	1	4	2	3	2	1	0	13	
	9	10	1	0	0	0	0	0	11	
	10	0	0	0	0	0	0	0	0	
	**Total**	11	5	3	6	7	4	2	**38**	**n/a**
ruvB	1	0	0	0	0	0	0	0	0	
	2	0	0	0	0	0	0	0	0	
	3	0	0	0	0	0	0	0	0	
	4	0	0	0	0	0	0	0	0	
	5	0	0	0	0	0	0	0	0	
	6	0	0	0	0	0	0	0	0	
	7	0	0	0	0	0	0	0	0	
	8	0	0	0	0	0	0	0	0	
	9	0	0	0	0	0	0	0	0	
	10	0	0	0	0	0	0	0	0	
	**Total**	0	0	0	0	0	0	0	**0**	**<0.045**
ruvA	1	0	0	0	0	0	0	0	0	
	2	0	0	0	0	0	0	0	0	
	3	0	0	0	0	0	0	0	0	
	4	0	0	0	0	0	0	0	0	
	5	0	0	0	0	0	0	0	0	
	6	0	0	0	0	0	0	0	0	
	7	0	3	1	1	0	5	3	13	
	8	0	0	0	0	0	0	0	0	
	9	0	0	0	0	0	0	0	0	
	10	0	0	0	0	0	0	0	0	
	**Total**	0	6	1	1	0	3	3	**13**	**0.1029**
mutL	1	0	0	2	14	0	0	0	16	
	2	0	0	0	0	0	0	0	0	
	3	0	0	0	0	0	0	0	0	
	4	9	0	0	1	4	0	1	15	
	5	0	0	0	0	0	0	0	0	
	6	0	0	0	0	0	0	0	0	
	7	0	0	0	0	0	0	0	0	
	8	0	0	0	0	0	0	0	0	
	9	0	0	0	0	0	0	0	0	
	10	0	0	0	0	0	0	0	0	
	**Total**	9	0	2	15	4	0	1	**31**	**0.3994**
recJ	1	0	0	0	0	0	0	0	0	
	2	0	0	0	0	4	0	0	4	
	3	0	4	0	0	0	2	0	6	
	4	0	0	0	0	0	0	0	0	
	5	0	0	0	0	5	0	0	5	
	6	0	0	0	0	0	0	0	0	
	7	0	0	0	2	0	0	0	2	
	8	3	0	0	0	0	0	0	3	
	9	0	0	0	0	0	0	0	0	
	10	0	0	0	0	0	0	0	0	
	**Total**	3	4	0	2	9	2	0	**20**	**0.6567**

Ear — Templated nucleotide changes, variable regions

Genotype	Clone	1	2	3	4	5	6	C	Total	P-value	
wt	1	0	0	0	0	0	0	0	0		
	2	1	0	0	0	14	0	0	15		
	3	0	1	7	1	0	5	2	16		
	4	1	0	0	0	0	1	0	2		
	5	0	0	2	2	0	0	1	5		
	6	3	5	6	4	17	1	1	37		
	7	0	4	1	1	0	1	0	7		
	8	12	0	1	0	0	6	1	20		
	9	1	0	0	2	1	0	0	4		
	10	0	0	0	0	0	0	0	0		**Total**
	**Total**	18	10	17	10	32	14	5	**106**	**n/a**	**314**
ruvB	1	0	0	0	0	0	0	0	0		
	2	0	0	0	0	0	0	0	0		
	3	0	0	0	0	0	0	0	0		
	4	0	0	0	0	0	0	0	0		
	5	0	0	0	0	0	0	0	0		
	6	0	0	0	0	0	0	0	0		
	7	0	0	0	0	0	0	0	0		
	8	0	0	0	0	0	0	0	0		
	9	0	0	0	0	0	0	0	0		
	10	0	0	0	0	0	0	0	0		**Total**
	**Total**	0	0	0	0	0	0	0	**0**	**<0.0014**	**0**
ruvA	1	0	0	0	0	0	0	0	0		
	2	0	0	0	0	0	0	0	0		
	3	0	0	0	0	0	0	0	0		
	4	0	0	0	0	0	0	0	0		
	5	0	0	0	0	0	0	0	0		
	6	0	0	0	0	0	0	0	0		
	7	0	0	0	0	0	0	0	0		
	8	0	0	0	0	0	0	0	0		
	9	0	0	0	0	0	0	0	0		
	10	0	0	0	0	0	0	0	0		**Total**
	**Total**	0	0	0	0	0	0	0	**0**	**<0.0014**	**13**
mutL	1	0	0	0	14	4	0	0	18		
	2	1	0	2	1	9	6	1	20		
	3	0	0	0	0	0	0	0	0		
	4	0	0	0	0	0	0	0	0		
	5	0	0	0	0	0	0	0	0		
	6	0	0	0	0	0	0	0	0		
	7	9	0	0	1	4	0	0	14		
	8	0	0	0	0	1	5	0	6		
	9	0	0	0	0	0	0	0	0		
	10	0	0	0	0	0	0	0	0		**Total**
	**Total**	10	0	2	16	18	11	1	**58**	**0.2113**	**166**
recJ	1	0	0	0	2	0	0	0	2		
	2	0	0	0	4	0	0	0	4		
	3	0	0	0	2	0	0	0	2		
	4	2	3	1	1	0	0	0	7		
	5	0	0	0	0	0	0	0	0		
	6	0	5	0	0	4	0	0	9		
	7	0	5	5	1	0	4	0	15		
	8	0	0	0	0	2	0	0	2		
	9	0	0	7	0	0	2	0	9		
	10	0	0	0	0	0	0	0	0		**Total**
	**Total**	2	13	13	10	6	6	0	**50**	**0.3403**	**200**

[a]Clones were sequenced from each tissue organ harvest culture taken at day 35

[b]C = constant regions otherwsie known as invariable regions, templated changes outside of variable regions that are traceable to specific cassettes

[c]p-values were calcualted using the Mann-Whitney student t-test
